# Wheat Bran Modifications for Enhanced Nutrition and Functionality in Selected Food Products

**DOI:** 10.3390/molecules26133918

**Published:** 2021-06-26

**Authors:** Oluwatoyin O. Onipe, Shonisani E. Ramashia, Afam I. O. Jideani

**Affiliations:** 1 Department of Food Science and Technology, Faculty of Science, Engineering and Agriculture, University of Venda, Thohoyandou 0950, South Africa; shonisani.ramashia@univen.ac.za (S.E.R.); afam.jideani@univen.ac.za (A.I.O.J.); 2 Postharvest-Handling Group, ISEKI-Food Association, 1190 Vienna, Austria

**Keywords:** wheat bran, valorisation, modification, flavour profile, hydration properties, microstructure, fibre solubilisation, functionality

## Abstract

The established use of wheat bran (WB) as a food ingredient is related to the nutritional components locked in its dietary fibre. Concurrently, the technological impairment it poses has impeded its use in product formulations. For over two decades, several modifications have been investigated to combat this problem. Ninety-three (93) studies (review and original research) published in English between January 1997 and April 2021 reporting WB modifications for improved nutritional, structural, and functional properties and prospective utilisation in food formulations were included in this paper. The modification methods include mechanical (milling), bioprocessing (enzymatic hydrolysis and fermentation with yeasts and bacteria), and thermal (dry heat, extrusion, autoclaving), treatments. This review condenses the current knowledge on the single and combined impact of various WB pre-treatments on its antioxidant profile, fibre solubilisation, hydration properties, microstructure, chemical properties, and technological properties. The use of modified WB in gluten-free, baked, and other food products was reviewed and possible gaps for future research are proposed. The application of modified WB will have broader application prospects in food formulations.

## 1. Introduction

The recommended dietary fibre (DF) ranges from 20–40 g/day [1,2]. CODEX Alimentarius defines DF as “carbohydrate polymers (derived from plant origin including fractions of lignin and/or other compounds associated with polysaccharides in the plant cell walls) with 10 or more monomeric units, which are not hydrolysed by the endogenous enzymes in the small intestine of humans and belong to the following categories: (a). Edible carbohydrate polymers naturally occurring in the food as consumed. (b) Carbohydrate polymers, which have been obtained from food raw material by physical, enzymatic, or chemical means and which have been shown to have a physiological effect of benefit to health as demonstrated by generally accepted scientific evidence to competent authorities” [3,4]. Other components in the definition of DF are shown in Table 1.

Regular consumption of DF improves the gastrointestinal microbiota through prebiotic function [5] and production of short-chain fatty acids through fermentation of the DF in the large intestine [6]. The health benefits attributed to this include a lowered risk of non-communicable diseases such as diabetes, obesity, cardiovascular diseases [7,8], and colon cancer [9]. An inverse relationship has been established between DF consumption (≥30 g/day) and reduction of the incidence of the diseases (Table 2). Fibre intake varies from one region of the world to the other, depending on factors such as age, gender, disease burden, economic development, and available food source [10].

**Figure 1:**
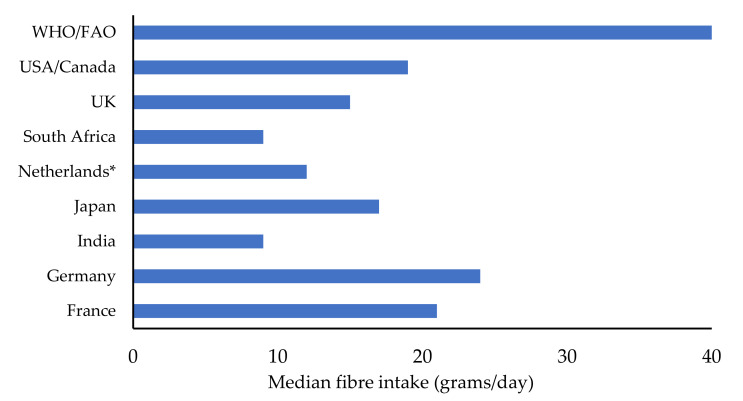
Mean fibre intake (g/day) of dietary fibre for adults in different countries of the world. Sources— [1,4,8,9,15,16] (* median fibre intake).

The reported maximum average fibre consumption from various food sources for adults (from age 18 and above) in various countries of the world is within the range of 9–24 g/day [1,4,8,15,16] and were all below the WHO/FAO recommended intake (Figure 1). This is an indication that fibre consumption from cereal grains, fruits, and vegetables is currently low and needs to be increased.

Bran of cereals such as wheat, rice, and corn are potential cheap raw materials that could be used in bakery products to improve their nutritional quality with minimal effect on consumer acceptability [17,18,19,20,21]. Wheat bran (WB) is a by-product of wheat grain milling and is a great source of dietary fibre up to 45 g/100 g, B-vitamins, minerals, and bioactive compounds [22,23]. Wheat bran has been found to be a beneficial application in animal feed and human food. The nutritional composition and health benefits of WB are linked to its fibre content made up of soluble dietary fibre (SDF) and insoluble dietary fibre (IDF). The SDF is mostly composed of resistant starch, lignin, and some hemicelluloses and cellulose, while IDF is made up of oligosaccharides, arabinoxylans, inulins, and celluloses [24]. Despite this rich nutrient load, the potential of WB is limited due to its poor suitability as a food ingredient enhanced by the presence of anti-nutrients (which forms complexes with minerals, thereby impeding bioaccessibility), sensitive chemicals (glutathione), endogenous enzymes (lipase, xylanase, amylase, and peptidase), and insoluble dietary fibre (IDF), which imparts negative technological effects on the quality of bakery products [25,26].

In recent years, WB has been subjected to several pre-treatments and modifications to improve its functionality and nutritional profile. These fibres have functional groups which react with other food molecules for optimum utilisation [27,28]. However, classic extraction and hydrolysis cannot adequately expose these groups or binding sites. Therefore, to advance the application of DF, many biological, chemical, and physical methods have been exploited to modify DF (composition and microstructure) from different food sources with anticipation of desirable effects on their physiological and functional properties. The link between WB consumption and better gut health, along with a lower risk of metabolic and cardiovascular diseases, has been established [23,25]. The impact of modified WB on human health (using animal and human studies) was recently reviewed by Deroover et al. [27]. Conflicting reports for the health benefits of WB modifications were highlighted; like size reduction, which had little or no effect on faecal bulking and lipid cholesterol like coarse bran [27]. Most of the studies to assess the health benefits of WB are quite old. Therefore, newer studies on the health benefits of the various WB modifications are recommended.

The use of WB in bakery products poses technological challenges because of its low gas holding and water binding capacity and poor dough viscosity of dough, thereby negatively impacting loaf volume and texture [25,28]. Cellulose and arabinoxylan in WB are known to be resistant due to their strong associations with other bran compounds and their high molecular weight [6]. Moreover, the IDF of WB is less hydrophilic, and thus impairs bread porosity, leading to denser texture and smaller loaf volume and height [28]. Therefore, any attempts geared toward increasing its hydrophilicity (the quality of a material or a molecule to be attracted to water molecules and tends to be dissolved by water) will improve its hydration, nutrient, and technological properties, accompanied by its impact on baked goods. This paper highlights the benefits and shortcomings of various pre-treatments and modifications to improve the nutritional profile and functionality of WB alone or in combination.

The literature search was conducted for articles published from 2000 to 2021, focusing on studies reporting modifications of wheat bran. Four electronic multidisciplinary databases were used to search for articles: Science Direct, SpringerLink, Google Scholar and Mendeley. Search keywords include “dietary fibre”, “wheat bran”, “autoclaved wheat bran”, “thermal treatment of wheat bran”, “wheat bran extrusion”, “cardiovascular diseases”, “coronary diseases”, “steam explosion”, and “milling”. Specific journals were also searched. Bibliographies from published review articles were also checked to supplement the electronic searches. About 249 articles were retrieved and screened based on their relevance to our study from their titles and abstracts: reducing the number of articles to 107. The final inclusion criteria were quality of the study, experimental design, statistical analysis, and reproducibility of the study. The full texts of the articles from the year 2002 to 2021 were finally reviewed, yielding a total of 93 articles.

## 2. Wheat Bran Modifications/Pre-Treatments

Modifications/pre-treatments such as thermal, enzymatic, and mechanical treatments geared towards reducing anti-nutritional content, extracting beneficial components, and improving solubility and functional properties of WB are discussed subsequently. The effect of various modifications on the functional and nutritional profile of WB is presented in Table 3.

### 2.1. Mechanical Treatment

The milling of WB increases its surface area, thereby increasing the bioaccessibility of nutrients. Various studies on the effect of size reduction of WB on its nutritional, functional, and microstructure have been investigated. Some of the quality properties of bran size reduction include reduced hydration properties [52,53] and phytic acid content [30]. On the other hand, the total phenolics, antioxidant activity [29,54], and water-soluble arabinoxylan content significantly increased by 26% due to milling [43,55]. The free TPC and specific phenolic acids in WB like cinnamic, sinapic, and vallinic acid increased up to 38% [43].

Ultra communition of WB from 400 to 16 µm caused a two-fold increase in the swelling capacity and three-fold increase in the DPPH and TPC contents [49]. Brewer et al. [29] noted that size reduction alone was not sufficient to improve the TPC of wheat bran as bound phenolics remained unchanged irrespective of their particle size. Therefore, it may be necessary to pair size reduction with other modification processes. In most studies where size reduction was paired with other pretreatment methods, milling preceded other processes such as hydrothermal and fermentation [30,37,46], and enzymatic treatment [39,45]. However, wet superfine grinding increased the surface area and strong water retention capacity of WB compared to native bran and thereby reduced the deleterious effect of WB on gluten development [56]. A combination of superfine grinding with steam explosion [40] increased hydration properties of WB and improved gluten development in the dough. This can be attributed to the introduction of moisture to the bran particles during the steam explosion treatment. The fat, ash, protein and SDF content of superfine WB fractions (<50 µm) increased compared to coarse WB. Applying another pretreatment method to milled bran improves its functionality because milling breaks down the particle size, thereby increasing the surface area and exposing the binding sites for further processing. However, there are limited studies on milling of pretreated WB. The application of fine WB fraction in bread production resulted in lower loaf volume, darker crumb, and a lower sensory score of bread [57], while the use of fine WB in the production of fried dough reduced glycemic index and had equal consumer acceptance scores with control sample [58,59]. Xu et al. [60] showed that the inclusion of superfine WB (39–435 µm) in dough increased the peak viscosity, water absorption, and starch hot-gel stability which resulted in a smaller specific volume of steamed bread. Preparation of noodles with up to 20% of superfine WB (27.9 µm) showed acceptable qualities comparable to the control noodle from refined wheat flour [61].

### 2.2. Thermal Treatment

#### 2.2.1. Dry Heat Treatment

Subjecting WB to dry heat treatment stabilizes it through the inactivation of enzymes which may cause rancidity and technological properties [62]. Examples of dry heat treatment include microwave, hot-air oven, roasting, and toasting [42,62,63]. Dry heat treatment is known to inactivate endogenous enzymes and reduce anti-nutritive heat-labile compounds such as PA, trypsin inhibitor, saponin, and oxalates [42]. Jacobs et al. [62] reported negative impacts of microwave and autoclave treatments as bran stabilisation methods. This is because microwaved bran was darker and burnt, with little effect on peroxidase activity, while autoclaving gelatinised the WB starch and caked the bran. However, a rinsing step would have resolved the cakiness. Meanwhile, Lauková et al. [34] reported a significant reduction in phytic acid content of WB microwaved at 800 W, for 2 min. Dry treatment in hot-air oven from 0 to 50 min significantly reduced peroxidase and hydration properties due to increased hydrophobicity in hot air-treated bran [62]. The hydration properties of a DF are linked to the ability of the fibre to interact, hold, and retain water in its pores [27,64]. The positive effect of dry heat treatment on the hydration properties of WB is linked to relaxation of bran macromolecules when it is hydrated and in turn has a high swelling capacity. The differences in the reports of these authors could be linked to varying methods and bran types used.

#### 2.2.2. Wet Heat Treatment

##### Autoclaving

Autoclaving is a wet thermal treatment that involves subjecting a material to high temperature under wet and pressurised conditions to sterilise or induce changes in the material [62]. Autoclaving treatment of bran is regarded as an effective dephytinization and fibre-modifying treatment [31]. The reduction of the free TPC and increased the amount of bound TPC in WB were reported (Table 3). The former could be because of the sensitivity of the phenolic compounds to heat which caused degradation and in turn a reduction [33]. The washing step after autoclaving could have also caused this reduction as the free TPC may have been leached into the slurry. However, Zhao et al. [32] reported a decrease in total TPC content of autoclaved WB. Where there was no change in phytic acid content in a study [33], others reported a significant reduction in the PA content of wheat bran [32]. Autoclaving alone may not be sufficient to reduce the PA content of WB due to the heat resistance of PA. However, lowering the pH of the bran matrix, then subjecting it to autoclaving increases the solubility and degradation of the PA-cation complex [30]. Increasing autoclaving time to 120 min in a low-pH bran system significantly reduced the PA content of WB.

##### Extrusion

Extrusion is a thermomechanical process that combines high/low thermal energy with other processes including mixing, shearing, size reduction, browning, texturizing, and shaping within a short time in an extruder to obtain a product with modified chemical, structural, and functional properties [24,65]. Extrusion mainly affects the WB fibre by increasing its solubility through mechanical rupture of the glycosidic bonds of the DF [66]. The thermal and shearing levels have been modified in several studies for maximum outcome in the extrusion process. This includes low/high temperature (30 to 180 °C) and shearing (60–400 rpm) at varying combinations [66,67,68]. The impact of extrusion on bran functionality and nutrition profile includes high IDF content and hydration properties [44], reduced mycotoxin and increased amino acid content [50], increased SDF at high temperature, and 45% feed-in moisture [66,68]. Although Gualberto et al. [69] reported no change in PA content, Kaur et al. [70] and Aktas-Akyildiz et al. [68] reported a maximum of 11.4% PA content of WB when extruded at 135 °C and a 16% moisture content. WB Extrusion caused a structural modification of the WEAX of WB through the introduction of new functional groups activated by oxidation [43]. Compared to non-extruded bran, there was no significant difference in the dough development time of dough with extruded WB; but a 19% increase in the volume of the bread baked from that dough with the help of an improver was observed. These differences are largely due to the presence of pre-gelatinised starch and soluble fibre in the latter [22]. Cookies enriched with extruded WB showed higher DF content and a 14% reduction in glycemic index content [71].

##### Steam Treatment

The comparative study of superheated steam (SS) and hot air (HA) treatment by Hu et al. [38] showed interesting differences between wet and hot air treatment. Total peroxidase was inactivated faster at 7 min by superheated steam than hot air at 16 min. Oxidative rancidity occurred in hot air but was avoided in superheated steam. Soluble phenolic compounds and sensory profile of WB treated with superheated steam were higher than hot air treatment. This positive effect of superheated steam may be attributed to higher moisture retention and solubilisation of fibre and conjugated phenolic acids. This was supported by the study of Aktas–Akyildiz et al. [39] where severe disruption of bran cell wall by steam explosion (steaming at 120–160 °C under pressure at 0.9–5 bar) was observed. Similarly, an increase in hydration, chemical, hydration, and antioxidant properties of steam explosion treatment of WB was observed [40]. The effect was enhanced when the steam-treated WB was milled into powder (≤75–425 µm), with the highest positive effect in the bran with lowest particle size. This is attributable to the increased hydrophilicity caused by the combined effect of heat treatment and milling. Steam explosion treatment (0.1–1.5 MPa, 110–196 °C) of WB disrupted the bran structure by breaking down the β-1,4 glycosidic bonds and reduced the contents of lignin, hemicellulose, and cellulose, thereby improving the potential use of WB in bakery products. Moreover, the intensity of sweet and fragrant flavours increased with an increase in the pressure of the steam treatment—a resultant effect of Maillard reaction [72]. This shows that steam explosion (optimal at 1.0 MPa) is a good modification method for amplifying good flavour compounds and reducing unfavourable flavour profiles of WB. Similarly, application of steam explosion (at 0.8 MPa, 170 °C, for 5 min) effectively inactivated endogenous enzymes in WB which led to a reduction of lipid oxidation and rancidity when WB was supplemented in wheat flour [41]. This improved the shelf life of the re-constituted flour, thus showing the potential for development of products with longer shelf life and improved quality attributes. Kong et al. [41] concluded that steam explosion effectively increased SDF, TFC, TPC and radical scavenging activity of WB by 27, 198, 83 and 21%, respectively.

### 2.3. Bioprocessing

#### 2.3.1. Fermentation

Fermentation is a process that involves exposing WB substrate to the fermentative effects (breakdown of complex sugars to simple sugars) of beneficial microorganisms to boost its potential. In recent years, several lactic acidbacteria have been used for solid-state fermentation of WB, intending to improve its technological, nutrient, and sensorial profile. Most of the bioactive compounds in WB are trapped in the layers of aleurone, pericarp, and testa [23]. The microorganisms that have been reported for fermentation of WB are yeasts (*Saccharomyces cerevisiae, Kazachstania exigua*) and bacteria (*Lactobacillus rhamnosus L. bulgaricus, Streptococcus thermophiles*, *L. brevis*). The fermentation of WB using lactic acid bacteria (LAB) acts on the fibre by metabolism of the conjugated phenolic compounds, breaking the linkage between them and the cell-wall polysaccharides, thus increasing the content of bound phenolic compounds [32,73]. During yeast fermentation of WB, a community of LAB and yeast cells is produced, thus making yeast a multifunctional starter to produce zymase and ethanol [47]. Zymase produced by yeasts causes structural degradation of the cell wall, thereby liberating the various antioxidant compounds. The ethanol produced during fermentation lowers the pH of the bran slurry, thereby exposing the yeast cells to oxidative stress. This in turn causes the cells to potentially produce protective mechanisms involving enzymatic antioxidation, thereby contributing to the antioxidative properties of the fermented bran [46].

A first step of autoclaving to de-activate indigenous enzymes and unwanted microorganisms which could impart negative properties during dough fermentation may be necessary [32]. An increase in free total phenolic content (TPC), water-extractable arabinoxylans (WEAX) and a decrease in phytic acid (PA) contents were noted at 24 and 48-h fermentation. A 60% increase in alkylresorcinols and up to a two-fold increase of SDF were observed in LAB and yeast fermented WB [32]. This had a resultant positive effect on the water holding capacity of WB, causing it to be more soluble in water (Table 3). An array of aroma compounds in fermented WB were reported and this could potentially improve the organoleptic properties and consumer acceptability of WB-enriched products [33]. Fermentation metabolites such as 2-hydroxyvaleric and 3-hydroxyphenyllactic acids which have anti-mycotoxigenic, antibacterial, and antifungal properties have been reported [32,33]. The acidification ability of LAB reduces the unpleasant flavour of WB, thus improving the sensorial properties [67]. The effect of the fermentation process has been enhanced through further modification like aeration of the fermentation chamber [74] and the addition of enzymes [67,75], particle size reduction [45], and extrusion [50].

#### 2.3.2. Enzymatic Modification

Enzymatic hydrolysis of WB can be achieved in three ways: (1) using commercially available enzymes, (2) fermenting the WB with microorganisms that produce the desired enzymatic action, and (3) activating endogenous enzymes present in WB. Since endogenous enzymes are concentrated in the layers of bran, it is only normal for them to be activated during the fermentation process [47]. Some of the known enzymes used for WB modification include phytase, xylanase, a-amylase, lipase, glutamic acid dehydrogenase β-glucanase, β-glucosidase, polygalacturonase, cellulase, β-endoxylanase, and α-L-arabinofuranosidase and endoglucanase [39,49,51,72,76,77,78]. The positive effects of enzyme treatment on WB include the release of bound phenolic acids, solubilisation of arabinoxylan, production of feruloylated oligosaccharides, increased mineral bioaccessibility, water-soluble antioxidant, SDF content, free amino acids, improvement of technological properties, enhanced flavour profile, reduced starch digestibility, and glycemic index [18,73]. The use of β-endoxylanase and α-L-arabinofuranosidase singly and in combination on WB (500 µm) increased its water retention capacity and TPC and DPPH content. Enzyme-treated WB improved the crumb texture profile and sensory properties and increased steamed bread volume, a resultant effect of altered water distribution in the dough because of WEAX xylooligosaccharide build-up [51]. The reduction of the phytic acid content of WB through yeast fermentation has been reported with positive outcomes. Servi et al. [30] reported a 96% PA reduction in WB subjected to an 8-h yeast fermentation. The pH reduction caused by the production of carbon dioxide and organic acids by yeast increases phytase activity thereby, increasing phytic acid solubility.

#### 2.3.3. Dephytinization Effects of Bioprocessing

Phytic acids (inositol polyphosphate) are undesirable anti-nutritive compounds naturally found in most grains, and they form complexes with minerals, thereby reducing their bioaccessibility in vivo [79]. The PA content of unprocessed WB is about 8% [54], which poses a challenge to mineral accessibility. Bran samples with a high content of dietary fibre and low PA content can be obtained through bio-processing methods. The hydrolysis of PA is usually achieved by phytase and phosphatase enzymes. De-phytinization increases postprandial mineral bioaccessibility. The negative effects of WB and rice bran on bread quality decreased significantly following dephytinization treatments. This study showed that fibre-enriched bread with a low PA content and an acceptable texture can be made using concentrated, dephytinized bran [26]. The PA content of WB decreased by 36% after a 48-h fermentation process with LAB [33] and 23–27% after fermentation with LAB and yeast [32]. Hydrolysis of PA during bioprocessing is possible in three (3) ways: (1) through the activity of the endogenous phytase of WB—this usually yields the highest degradation rate of about 90% [80], (2) activity of the phosphatase and phytase from the microorganisms used in fermentation, and (3) acidification of phytase for its optimum hydrolytic potential is strongly pH-dependent (pH 5) during fermentation [32].

### 2.4. Combination of Pre-Treatment Methods

#### 2.4.1. Enzymatic and Fermentation Treatment

While one valorisation method may yield some results, tremendous results have been reported when one or more treatments were used in combination or sequentially. Arte et al. [75] demonstrated the importance of the combined effect of enzymatic treatment and microbial fermentation to exponentially boost the nutritional profile of WB. This led to an increase in total phenols, PA degradation, protein solubilisation, and digestibility through the activation of endogenous protease activity. Similarly, a combination of fermentation (by yeasts and LAB) and enzymatic treatment (xylanase and amylase) led to in-vitro catabolism of DF and ferulic acid, a release of protein from aleurone, increased solubilisation of bran proteins, and modification of bran microstructure changes in bran microstructure [81].

#### 2.4.2. Thermomechanical and Bioprocessing

A sequential combination of mechanical (size reduction), fermentation with microorganisms and enzymatic treatments (xylanase, endoglucanase, α-amylase, and β-glucanase) was carried out by Coda et al. [45,82]. Fermentation with *Lactobacillus brevis* and *Kazachstania exigua* at 20 °C for 24 h caused up to a six-fold significant (*p* < 0.05) increase in WEAX and exponentially increased to 11.5-fold when fermentation was combined with enzymatic treatment. Similarly, peptide content increased from 34 to 81 mg/kg in lowest bran fraction (50 µm) subjected to fermentation and enzymatic treatment [45]. Milling exposed and disintegrated the intact cell layers (composed of IDF, xylan and lignin). Further bioprocessing of milled bran with LAB and enzymes degraded the glycosidic bond of the cell walls, leading to an increased antioxidant content. The sequential combination of extrusion technology and a 4 h enzymatic hydrolysis sequentially enhanced the SDF content of WB more than the single effect of each treatment [66]. Similarly, Bartkiene et al. [50] demonstrated the potential of simultaneous combination of extrusion (115 and 130 °C for 16–25 rpm screw speed) and fermentation (30 °C) with selected LAB strains (*L. uvarum* & *L*. *plantarum*) in the reduction of mycotoxins and improvement of biogenic amine content. Compared to untreated WB, the lowest mycotoxin level (29.8 µg/kg)—an 80% reduction—was found in sample extruded at maximum conditions (130 °C at 25 rpm) fermented with *L. uvarum* strain. The free amino acid content increased mostly after fermentation because LAB promotes proteolysis through their endogenous proteases [50].

The combination effect of milling, steam explosion, and enzymatic hydrolysis increased the WEAX content of WB. The positive attributes incurred because of these three treatments can be explained as such: grinding and steam explosion caused a breakdown of the cell wall, thereby increasing the surface area and the binding sites accessible to the enzymes [39]. In a recent study, size reduction (100–400 µm), autoclaving, and yeast fermentation were used for WB modification. The physicochemical properties of WB were enhanced in fermented samples with different ranges in the various particle sizes, while autoclaving increased IDF content only. Phytic acid was well degraded by fermentation and the lowest content was found in the smallest particle size at 62% reduction [83]. Aktas-Akyildiz et al. [68] combined extrusion and enzymatic treatment to modify WB. Extrusion of WB was first carried out in an extruder using 100/200 rpm screw speeds at moisture contents of 12, 14, and 16%, at the exit-die temperatures of 105, 120, or 135 °C. Thereafter, the extruded bran was enzymatically treated with hemicellulose. Extrusion (high shear, low moisture and temperature) increased the SDF content from 2.3 in native bran to 5.3%. This was achieved by the conversion of IDF to SDF. Although enzymatic treatment of extruded bran increased SDF content further, the increase was not statistically significant. Modifications targeted at a specific nutrient can be achieved by selective and specific treatments. Ferri et al. [84] were able to achieve a 40-fold recovery of ferulic acid by sequential treatment of WB by autoclaving/steam explosion at a water/bran ratio of 20:1 (for bran rehydration), followed by enzymatic treatment with termamyl and alcalase to hydrolyse sugars and protein; and a final enzymolysis with ferulyol and pentopan for solubilisation of phenols.

From the various pre-treatment combinations discussed, it is evident that there were more studies on mechanical/thermal modification before bioprocessing. Generally, combination treatments showed better nutritional properties than single treatments. Only a handful of the studies utilised the pre-treated WB in food formulations; therefore, such studies are recommended.

## 3. Modified Wheat Bran as a Functional Ingredient in Selected Food Products

### 3.1. Bread

Bread produced from dough supplemented with fermented WB had better loaf volume, softer crumb, and reduced staling compared to non-fermented WB [47,82,83]. The folate content of breads supplemented with fermented WB had 32–62% higher folate content [47]. Breads produced from fermented bran had a better shelf life. This is probably because fermented bran retards starch retrogradation and alters water distribution between starch and gluten in the bread crumb [76]. The effectiveness of bioprocessing on improved bread properties can be linked to lower pasting viscosity of starch, solubilization of arabinoxylans, improved rheology, and faster carbon dioxide production [48,85]. The development time of dough with pre-fermented WB (3.9 min) was higher than wheat flour (2.8 min), but was significantly lower than unfermented WB (5.8 min). The specific volume of bread with 20% fermented WB was comparable to control. This shows the potential of fermentation to improve rheological properties of dough and resulting bread characteristics [48]. The use of bran treated with enzymes and/or steam explosion increased the specific volume, SDF, and reduced hardness and PA contents of the breads compared to the reference bread [39].

### 3.2. Cookies and Cakes

The utilisation of heat-stabilized WB (using microwave and hot air) in cookie production improved the spread ratio and reduced hardness compared to cookies with untreated bran. Moreover, the cookies enriched with stabilized bran were more acceptable for assessors at a 5% incorporation level [86]. The specific volume and firmness of cake made from small particle sizes of WB (50 and 80 µm) increased significantly up to 24% substitution level in the cake batter formulation. Sensory scores of cakes with 24% WB substitution were not significantly different from control cake. This implies that acceptable fibre-rich cakes can be produced from incorporation of ≤20% of small particle size of WB in batter formulations [87].

### 3.3. Noodles and Pasta

Incorporation of 30% superfine WB (27.9 µm) reduced hardness (%) and increased adhesiveness of cooked wheat noodles. The appearance, taste, smell, and palatability of the noodles were comparable to control (no bran) noodles at 20% milled bran incorporation [61]. Steam treatment increased stability of WB during storage (90 days), reducing lipase activity by 50%. Substitution of 40% of heat-treated WB in pasta formulation reduced cooking loss by 27%, increasing TDF five-fold with higher sensory scores compared to control pasta [88]. Chen et al. [89] concluded that production of fibre and sensorially acceptable Chinese noodles was possible by substituting 5–10% fine bran (210 µm) or 5% medium bran (530 µm) in wheat flour.

### 3.4. Fried Dough

The utilisation of milled WB of various particle sizes (6.87, 200, 250, and 500 µm) in deep-fried dough product formulations reduced fat content ranging from 2.7% to 44% [58,90] and glycemic index [59] depending on the level of addition, ranging from 1 to 20%.

### 3.5. Gluten-Free Products

The appreciably high nutrient contents of WB may be exploited for use in development of gluten-free products, but its gluten content (110 g/kg) may hinder that [91]. Therefore, gluten degradation has been carried out using peptidase enzyme. Enzymatic attempts to degrade gluten content of plant products were adequately reviewed by Scherf et al. [91]. These enzymes can be gotten from plant (cereal germination), insects (*Rhizopertha dominica*), fungal (*Aspergillus* sp.), bacterial (*Bacillus* and *Lactobacillus* spp.), and/or genetically engineered. Walter et al. [92] demonstrated the gluten degradation of raw, germinated, and commercial WB using a proline-specific peptidase from *Aspergillus niger*. Gluten degraded below limit of quantification at enzyme quantity and incubation time of 400 mg/mL, and 48 h incubation time and total degradation at 750 mg/mL enzyme quantity at 72 h incubation. The highest degradation was observed in the WB from germinated wheat due to initial partial degradation caused by the peptidase produced during the germination process before enzymatic treatment. The degradation of gluten in a bread drink was possible after 20 min of incubation with peptidase [92]. Recently, Tanasković et al. [93] carried out a solid-state fermentation of WB with *Bacillus* sp. TMF-2. The fermented bran had three times the soluble phenolic content of the raw bran. The free radical scavenging rate to reduce Fe3+ increased by 10-fold. More importantly, after 168 h of fermentation, simultaneous increase in protease alongside a significant reduction in gluten content (r = −0.80) was observed. The ability of protease to degrade gluten resulted in gluten-free wheat bran. There is a need for more studies on the utilisation of gluten-degraded WB in the development of gluten-free food products.

## 4. Conclusions

The technological impairment caused by WB is a known fact and several methods to combat this have been discussed in this paper. The enhanced properties in modified WB depended on the processing method, extraction, and analysis method used which differed from one study to the other. This implies that the anticipated outcomes will determine the prospective modification methods to be used. Bioprocessing tremendously dephytinized WB, improving its antioxidant and flavour profile. Autoclaving and grinding reduced phytic acid significantly, superheated steam deactivated endogenous enzymes quickly, and extrusion increased solubility, modifying the structure of arabinoxylan. Although dry heat treatment was unfavourable for phytochemicals in WB, recent efforts in steam explosion showed good promise for the production of modified WB with improved antioxidant activity, flavour profile, shelf life, and chemical composition. A combination of pre-treatments showed promising results for the creation of functional WB with an improved nutritional profile. However, there are sequential combination treatments that have not been used, thus requiring more research. Only a fraction of the studies reviewed in this paper used the modified WB for food enrichment. Hence, follow-up studies on the use of modified WB as a functional food ingredient remain an open research prospect.

## Figures and Tables

**Table 1:** Some examples of components included within the CODEX definition of fibre and the food sources.

Fibre Component	Description	Food Sources
Cellulose	Polysaccharides comprising up to 10,000 closely packed glucose units arranged linearly.	Grains, vegetables, fruit, nuts, cereal bran.
Hemicellulose	Polysaccharides containing sugars other than glucose.	Cereal grains, vegetables, fruit, legumes (for example peas, beans, chickpeas, lentils) and nuts.
Lignin	A non-carbohydrate component associated with plant walls.	Foods with a woody component, for example, celery and the outer layers of cereal grains.
Beta-glucans	Glucose polymers that (unlike cellulose) have a branched structure	Mainly found in cell wall of oats and barley.
Pectins	A non-starch polysaccharide common to all cell walls.	Fruits and vegetables, legumes, nuts, and potatoes.
Gums and mucilages	Non-starch polysaccharides are thick gel-forming fibres that help hold plant cell walls together.	Gums: seeds and seaweed extracts; Mucilages: pysillium seeds. Gums and mucillages are used as gelling agents, thickeners, stabilisers, and emulsifying agents.
Resistant starch	Starch and the products of starch digestion that are not absorbed by the small intestine.	Legumes, potatoes, cereal grains
Oligosaccharides	Short-chain carbohydrates of 3–9 monomers. These include fructo-oligosaccharides and galacto-oligosaccharides.	Onions, chicory, Jerusalem artichokes
Micro components (waxes, cutin and suberin)	Micro components of the plant structures.	Cereal grains

Source: British Nutrition Foundation (2018).

**Table 2:** Summary of the findings of the meta-analyses of cohort studies investigating inverse relationships between fibre consumption and specific health outcomes.

Disease	Number of Studies in Meta-Analysis	Findings	Reference
Cardiovascular disease	10	Inverse association—RR of 0.91 (95% CI 0.88–0.94) for each 7 g/day increase at *p* < 0.001	[11]
Coronary events	12	Inverse association—RR 0.91 (95% CI 0.87–0.94) for each 7 g/day increase at *p* < 0.001	[11]
Stroke	7	Inverse association with incidence of haemorrhagic plus ischemic stroke RR 0.93 (95% CI 0.88, 0.98) for each 7 g/day increase at *p* = 0.002	[12]
Colorectal cancer	8	Inverse association with the incidence of colorectal cancer RR = 0.88 (95% CI 0.83–0.97) for each 10 g/day at increase; *p* < 0.05.	[13]
Type 2 diabetes	10	Inverse association RR 0.87 (95% CI 0.90, 0.97) for each 7 g/day increase; *p* = 0.001	[14]

RR—relative risk, CI—confidence interval.

**Table 3:** Wheat bran modifications and their impact on functionality and nutritional properties.

Modification Type	Impact on Functionality	Effect on Nutritional Properties	Reference
Thermomechanical Treatment			
Milling (900, 750, 500 and 355 µm)	ND	Bound total phenolic content (TPC) and total flavonoid increased by 1.5-fold, total anthocyanin by 2-fold. Zeaxanthin and beta carotene increased in medium bran and lutein in fine bran fraction. Milling did not affect the DPPH content of wheat bran (WB).	[29]
Autoclaving (121 °C for 0.5–2 h pH: 3.5–6.2)	ND	At native pH (6–6.2) no change in phytic acid (PA) occurred. Maximum reduction * (96%) at pH 3.5 and 2 h autoclaving was reported.	[30]
Autoclaving (121 °C for 0.5–1.5 h, pH: 3.5–6.6)	ND	A 96% decrease * of PA at pH 4 and 1 h processing time. Significant increase in insoluble dietary fibre (IDF) and soluble dietary fibre (SDF). Autoclaving increased the bound and total TPC of wheat bran.	[31]
Autoclaving conditions (121 °C for 20–21 min)	Increase in water retention capacity (WRC) and water holding capacity (WHC). No change in microstructure.	Reduction in TPC, PA and IDF. An increase in alkylresorcinol, water-extractable arabinoxylan (WEAX) was observed.	[32,33]
Microwave (800 W, 2 min) and hot air oven, (150 °C for 20 min)	Water absorption capacity and swelling capacity (SC) markedly increased in both treatments by up to 11%. Hot air treatment increased bran lightness.	Both methods increased protein and total dietary fibre content. A decrease in moisture and PA content was also observed	[34]
Extrusion (temperature: 80 and 120℃, screw speed: 120 and 250 rpm)	ND	Extrusion increased WEAX content, SDF content. Fermentable carbohydrates and short-chain fatty acid (SCFA) content were higher in extruded bran.	[35]
Extrusion (temperature:140 °C, screw speed: 150 rpm, 45% moisture) + size reduction (830, 380, 250 and 180 µm)	The surface of extruded bran was full of holes and had an irregular surface structure. WHC < ORC and SC increased with extrusion and size reduction	SDF of extruded WB increased by 70% *. Antioxidant properties increased as dosage (mg/mL) increased.	[36]
Extrusion (temperature: 120 and 145 °C, moisture: 23, 27 and 33% screw speed: 310 rpm)	A greater extent of degradation of pericarp and aleurone layer of WB was caused by very high shear than low shear extrusion using light microscopy.	A 1.8-fold and 3.5-fold increase in WEAX and free ferulic acid. PA content decreased by 19% * andA small increase in SCFA was reported after 48 h fermentation.	[6]
Milling (420, 280, 170 and 90 µm) Hydrothermal (acetate buffer (pH 4.8) at 55 °C, 60 min and incubation at 5 5°C for 24 h) Yeast fermentation (8 h at 30 °C)	Reduced WHC and swelling power and increase in water solubility index of fermented and hydrothermal bran. Size reduction increased L * values of WB.	34, 57 and 76% reduction * in PA content in milled, fermented, and hydrothermal WB. Hydrothermal and fermentation treatments increased the total dietary fibre (TDF), SDF and reduced the IDF content of WB. Mineral contents reduced with all treatments.	[37]
Super-heated steam (15.0 m^3^/h, 170 °C for 20 min) Hot air processing in an electro-thermostatic blast oven (170 °C for 20 min)	ND	Superheated steam was more efficient in enzyme inactivation, enhancement of non-starch nutrients, reduction of peroxide value, higher soluble phenolic content, and better sensory profile than hot air treatment.	[38]
Milling + Steam explosion (120–160 °C for 5–10 min)	Lightness values of WB treated with steam explosion decreased. Severe disruption of bran cell wall by grinding and steam explosion was reported.	Milling and steam explosion alone and in combination increased AX solubilisation in fine bran. Loaf volume, SDF increased, and PA content reduced in breads with pre-treated WB.	[39]
Steam explosion (0.8 MPa, 170 °C, 5 min) + grinding (425–75 µm)	Steam explosion and milling increased WB porosity, WHC and SC.	Fat, starch, protein, SDF, TPC, total flavonoids and DPPH contents increased with steam explosion and size reduction.	[40]
Steam explosion (0.3, 0.5 & 0.8 MPa, at 170 °C, for 5 min)	Lipase and peroxidase activity reduced and shelf life increased.	Protein, and lipid content remain unchanged. SDF, TPC, TFC and DPPH values increased at maximum steam (0.8 MPa).	[41]
Microwave (2450 MHz at 1.5–2.5 min) Hot air oven (100 & 110 °C, 15, 20 & 25 min) Steaming (100, 110 & 115 °C, 15, 20 at 25 min)	All treatments increased bulk density and darkened the bran samples.	Microwave treatment at 2.5 min caused a significant reduction of PA, polyphenols, saponins, trypsin inhibitors and toxicants	[42]
Milling (ultra-centrifugal mill-500 µm) + Extrusion	Structural modification of WEAX was more distinct in extruded bran.	Milling increased WEAX content (26% *) and reduced molecular weight of WB. No significant change in TPC, but 38% * increase in free TPC of milled bran.	[43]
Milling + Extrusion	About 1.5-fold increase in WHC and IDF content of bran fractions and a decrease in SDF content after extrusion process.	Antioxidant capacity increased as the particle sizes of the milled bran reduced up to 180µm.	[44]
**Bioprocessing (Fermentation and Enzymatic Treatments)**			
Fermentation at 2–8 h with *Saccharomyces. cerevisiae* (3–9%).	ND	A reduction (≤96%) in phytic acid content with an increase in fermentation time and yeast concentration.	[30]
*Lactobacillus brevis* and *Kazachstania exigua* (20 °C for24 h) + enzymes (xylanase, endoglucanase and β-glucanase)	Partial degradation of bran cell wall.	A sixfold increase * in WEAX in fermented bran and up to 11.5-fold increase * when fermentation was combined with enzymes. A 50% increase * in peptide content was observed in bioprocessed bran compared to native bran	[45]
Fermentation with *L. rhamnosus* (37 °C for 24–48 h)	ND	Free TPC and WEAX increased significantly. Caffeic acid was notable in fermented bran. A reduction in phytic acid (PA) content was observed.	[33]
Fermentation with *S. cerevisiae* (30 °C for 6 h)	Increase in water absorption capacity of WB.	An 86% decrease * of PA at pH 4 and 1 h processing time. TDF of bran was not affected by fermentation.	[26]
Fermentation with *S. cerevisiae* at (30 °C for 6 days)	ND	A significant increase in the TPC, DPPH, antioxidant activity of WB was observed on day 3 of fermentation.	[46]
Spontaneous and yeast fermentation (20 & 32 °C for 20 h)	ND	Significant increase (≥40% *) in folates, free ferulic acid and soluble AX in yeast-fermented bran. Acidification of bran slurries at maximum fermentation temperature.	[47]
Fermentation with *L. brevis* (28 °C for 16 h)	An increase in gas retention of dough and bread volume was observed with the inclusion of fermented WB. Significant reduction in bread staling compared to bread with unfermented WB. Improved viscoelasticity of dough	There was a two- and four-fold increase * in WEAX and SDF of fermented WB compared to native bran.	[48]
Enzymatic treatment (cellulase and xylanase)	WRC increased by 16%. Enzymatic treatment improved oil holding and swelling capacity. Glucose adsorption capacity improved by 1.4-fold. Loose structure, wall structure damaged/degradation of wall PS.	A twofold increase of TPC, and antioxidant properties of enzyme-treated WB compared to the control sample.	[49]
Treatment with *Lactobacillus bulgaricus, Streptococcus* *thermophiles and Saccharomyces cerevisiae* (alone and in combination)–37 °C for 24 h & 48 h	The WHC and WRC improved significantly in fermented WB. Partial degradation of aleurone cells.	Five-fold increase in WEAX content, 60% increase in phenolic lipids, 2-fold increase in SDF, 23–27% reduction in PA.	[32]
Extrusion (115 and 130 °C; screw speeds: 16, 20, and 25 rpm) + fermentation (*L. plantarum* and *L. uvarum*)	ND	Combination of both treatments lowered mycotoxin content by 80.6% * and increased biogenic amines by 42.9% * of bran samples. Fructose content increased by 15% * after fermentation.	[50]
Enzymatic treatment (β-endoxylanase and α-L-arabinofuranosidase)	WRC and fat binding capacity increased in single and combined enzyme-treated WB. Improved porosity of enzyme-treated bran dough	TPC and DPPH content increased in single and combined enzyme-treated WB. pH reduced in WB treated with xylanase and combined enzymes.	[51]

ND—not determined, *—statistically significant (*p* < 0.05).
